# Creation and Plasmon-Assisted Photosensitization of Annealed Z-Schemes for Sunlight-Only Water Splitting

**DOI:** 10.1021/acsami.3c02884

**Published:** 2023-06-06

**Authors:** Denis Zabelin, Kamil Severa, Jaroslav Kuliček, Bohuslav Rezek, Anastasiia Tulupova, Roman Elashnikov, Anna Zabelina, Vasilii Burtsev, Petr Sajdl, Elena Miliutina, Vaclav Svorcik, Oleksiy Lyutakov

**Affiliations:** † Department of Solid State Engineering, University of Chemistry and Technology, 16628 Prague, Czech Republic; ‡ Faculty of Electrical Engineering, Czech Technical University in Prague, 16627 Prague, Czech Republic

**Keywords:** Z-scheme, plasmon photosensitization, artificial leaf, overall water splitting, sunlight

## Abstract

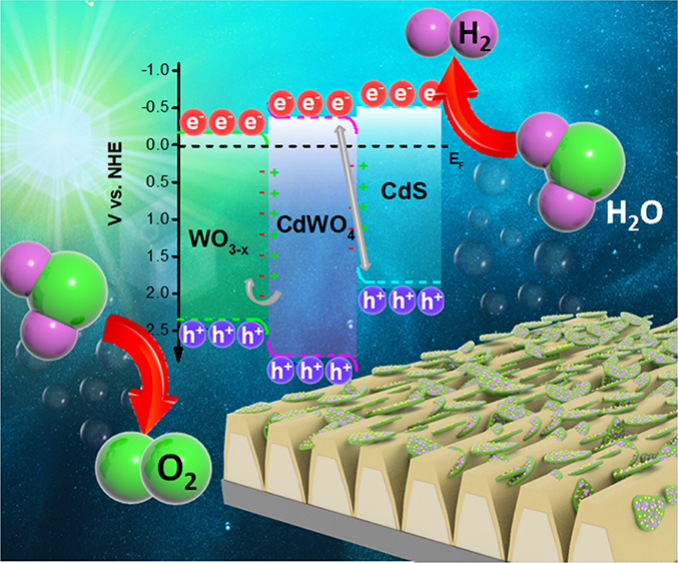

Solely light-induced water splitting represents a promising avenue for a carbon-free energy future, based on reliable energy sources. Such processes can be performed using coupled semiconductor materials (the so-called direct Z-scheme design) that facilitate spatial separation of (photo)excited electrons and holes, prevent their recombination, and allow water-splitting half-reactions proceeding at each corresponding semiconductor side. In this work, we proposed and prepared a specific structure, based on WO_3g–*x*_/CdWO_4_/CdS coupled semiconductors, created by annealing of a common WO_3_/CdS direct Z-scheme. WO_3–*x*_/CdWO_4_/CdS flakes were further combined with a plasmon-active grating for the creation of the so-called artificial leaf design, making possible complete utilization of the sunlight spectrum. The proposed structure enables water splitting with high production of stoichiometric amounts of oxygen and hydrogen without undesirable catalyst photodegradation. Several control experiments confirm the creation of electrons and holes participating in the water splitting half-reaction in a spatially selective manner.

## 1 Introduction

The most significant challenges of the beginning of the 21st century are closely related to the need for renewable and carbon-free energy sources that can ensure energy safety and, at the same time, do not contribute to global warming.^1,2^ In this sense, the direct utilization of sunlight energy for water photolysis and the generation of green hydrogen can be considered as one of the most promising avenues.^3−5^ Therefore, considerable attention is paid to the design and creation of different materials that can efficiently absorb sunlight and ensure effective water photolysis.^6−8^ In particular, countless efforts were devoted to the design of highly efficient and stable photoelectrochemical systems, implementing different semiconductor materials, such as metal oxides, sulfides, and nitrides, as well as several ternary compounds.^9−15^ The main attention has been focused on the increase of material efficiency in light-induced water splitting through facet and interface engineering, the introduction of vacancies, cocatalyst loading, etc.^16−22^ However, it is difficult with a single material to achieve proper combination of redox activity sufficient for water splitting, positions of the valence and conductive bands (VB and CB), and sufficient sunlight absorption.^23,24^ As an elegant solution, the so-called Z-scheme or S-scheme designs, based on coupled semiconductors with suitable positions of the VB and CB and Fermi level(s) were proposed.^25−32^ Light absorption in both coupled semiconductors leads to excitation of higher and lower redox-active electron(s) and hole(s).^33−35^ Electrons and holes with higher redox activity participate in water splitting (or in consumption of added sacrificial agents), while residual electrons and holes recombine.^36−38^

Several combinations of semiconductors, with a suitable CB and VB position and surface redox activities have been reported for the design of the Z-scheme.^39−47^ The possibility of photogenerated carrier separation and increasing material photostability make Z-scheme-based water photolysis more and more attractive.^2,16,17,48,49^ The main drawback of the common Z-scheme consists in the contact interface between two materials and possible lattice mismatches and interface defects, which can prevent efficient charge carrier transfer between materials.^50^ In addition, insufficient redox activity can often lead to the oxidation or reduction of one semiconductor in the Z-scheme (for example, the oxidation of S^2–^ in the common WO_3_/CdS structure). This so-called photodegradation may lead to a significant decrease in water-splitting efficiency, most often observed as the appearance of nonstoichiometric amounts of oxygen and hydrogen during water splitting.^51^ To overcome these drawbacks and to facilitate charge transport in a stepwise manner, the combination of more than two semiconductors has recently been proposed.^50,52−57^

In this paper, the preparation and use of the annealed Z-scheme (WO_3–*x*_/CdWO_4_/CdS), developed from the common direct Z-scheme (WO_3_/CdS)^58^ is described. To compensate lattice mismatch between WO_3_ and CdS, an interface CdWO_4_ layer was created between WO_3_ and CdS by thermal annealing. The CdWO_4_ layer also provides additional pathways for charge transfer between CdS and WO_3_. The layer formation was also accompanied by the appearance of redox-active oxygen vacancies in WO_3._^59,60^ In the next step, we performed photosensitization of the created Z-scheme by deposition of WO_3–*x*_/CdWO_4_/CdS on the surface of plasmon-active grating. It was expected that plasmon photosensitization can efficiently enhance the redox activity of the created material(s) and clear the way for the use of the NIR part of the sunlight spectrum.^61−63^ Finally, solely light-induced water splitting was demonstrated in the so-called “artificial leaf” manner with spatially selective high production of stoichiometric amounts of oxygen and hydrogen without material photodegradation.

## 2 Results and Discussion

### 2.1 Main Structure Design and Preparation Route

**Figure 1:**
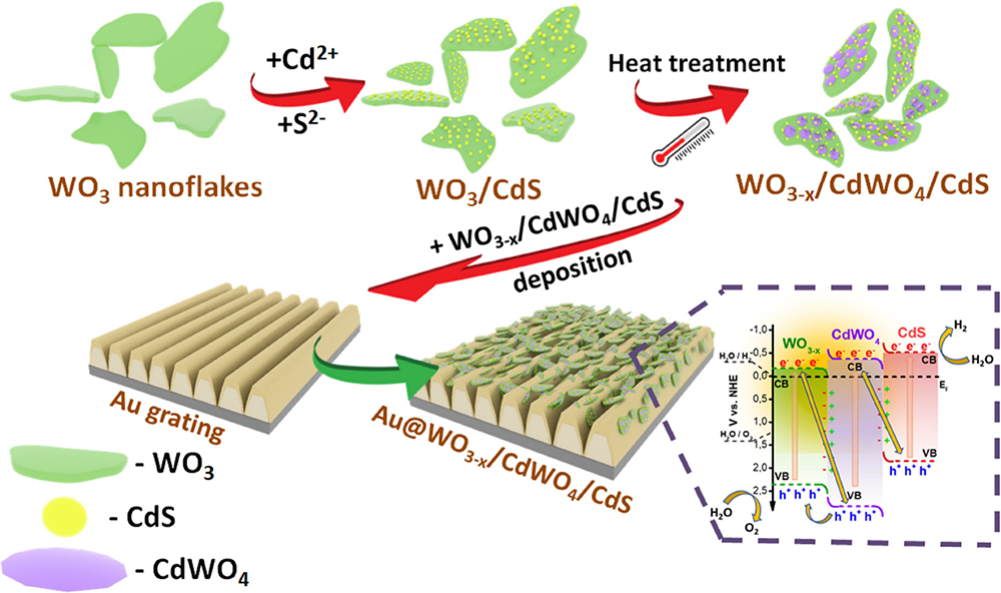
Schematic representation of the preparation of the annealed Z-scheme (with additional CdWO_4_ layer formation between CdS and WO_3–*x*_) and its subsequent coupling with the plasmon-active gold grating.

The creation of the proposed Z-scheme and its coupling with plasmon-active grating are presented schematically in Figure 1. First, we performed the surface-assisted synthesis of CdS nanostructures on WO_3_ flake surfaces. Then, the WO_3_/CdS coupled semiconductors were subjected to thermal annealing (samples are further designated as WC–X, where X is the annealing temperature) with the aim of performing solid state synthesis of the CdWO_4_ interface layer between the coupled semiconductors. At the same time, a plasmon-active grating surface was prepared by depositing a thin layer of gold (20 nm) on a periodically modulated polymer template. In the next step, WO_3–*x*_/CdWO_4_/CdS flakes were deposited on the surface of the plasmon-active grating in order to achieve light-induced charge generation and separation with possible participation of long-wavelength photons in water splitting.

### 2.2 Optimization of the Annealing Temperature

**Figure 2:**
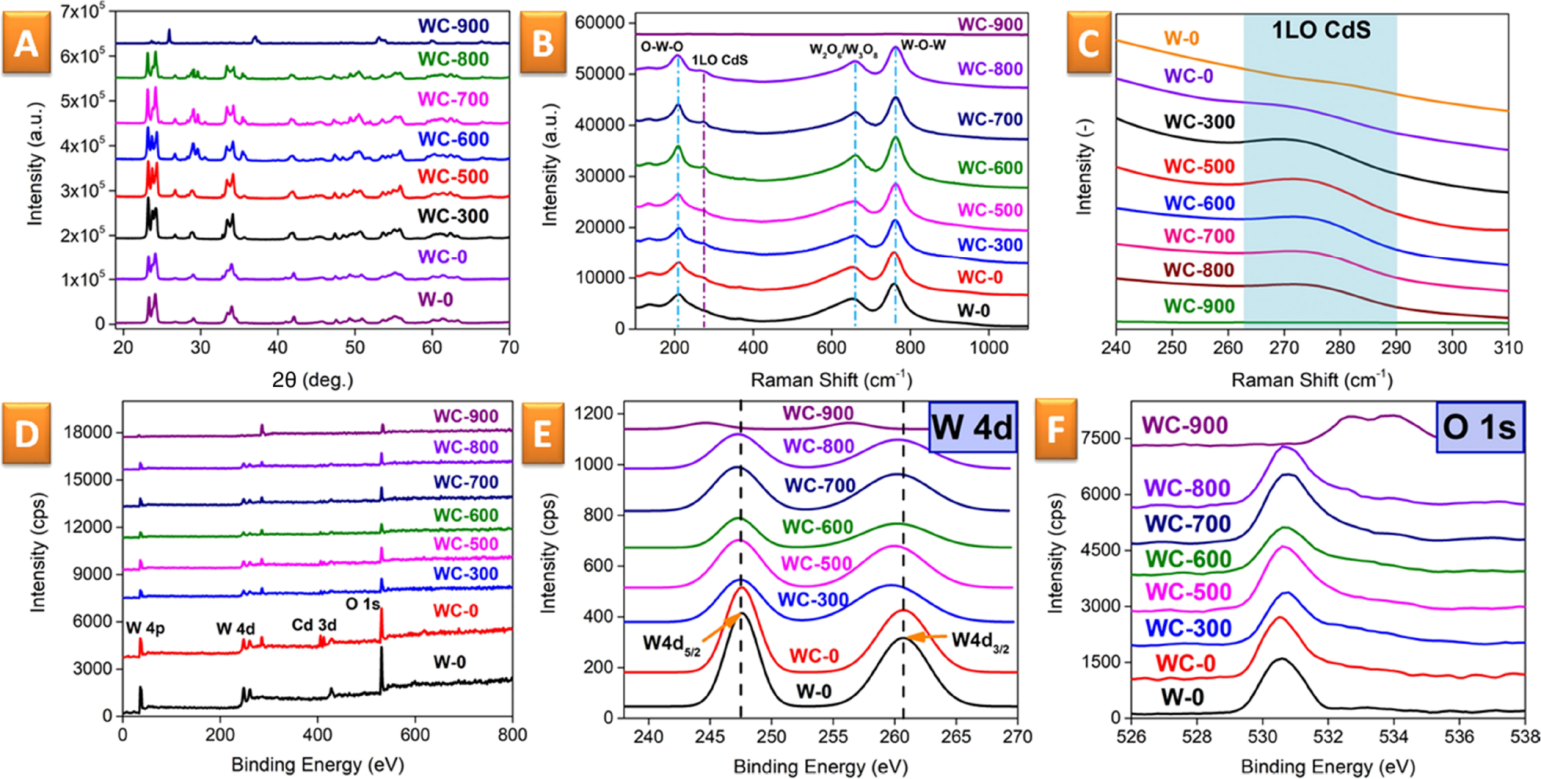
Evolution of the WO_3_/CdS: XRD pattern (A) and Raman spectra (B) as a function of the annealing temperature; (C) highlighted Raman spectral area with characteristic signals from the crystalline CdS phase as a function of the annealing temperature; (D) raw XPS spectra of pristine and annealed samples; and (E, F) details of characteristic W 4d_3/2_ XPS and O 1s peaks.

Annealing was carried out at different temperatures in the 300–900 °C temperature range and the obtained materials were analyzed by X-ray diffraction (XRD), X-ray photoelectron spectroscopy (XPS), Raman spectroscopy, and high-resolution transmission electron microscopy (HRTEM) techniques. In the case of XRD measurements (Figure 2A), the obtained patterns reveal the appearance of some additional reflexes at 26.7° (002), 28.3 (101), and 43.9 (110), which are attributed to the crystallization of CdS (ICDD 04-006-3897) during annealing. It is also clearly seen that all samples contain some crystalline WO_3_ phase, which dominate in all patterns. When increasing the annealing temperature, the phase transition is observed from monoclinic ε-WO_3_ (23.2° (002) and 21.1° (110)—ICDD 01-087-2399) to monoclinic WO_3_ (24.4° (200), 23.6° (020), and 23.1° (002)—ICDD 01-083-0950). However, starting from 600 °C, we also observe the characteristic reflexes of the CdWO_4_ phase (29.0° (111) and 29.6° (111)—ICDD 01-073-6298). Furthermore, annealing also results in the appearance of additional reflexes (26.8°, 29.1°, and 35.5°), characteristic for the creation of oxygen vacancies in initially stoichiometric WO_3_ (i.e., formation of the WO_3–*x*_ phase, ICDD 01-084-1516). The characteristic reflexes of CdWO_4_ and WO_3–*x*_ appear after annealing at a temperature above 600 °C and become clearly evident after annealing at 700 °C. On the other hand, when the sample is annealed at 900 °C, the appearance of a WO_2_ phase with low crystallinity was observed (ICDD 04-003-2345). Therefore, based on the XRD results, the annealing at 700 °C appears to be optimal for the formation of WO_3–*x*_/CdWO_4_/CdS flakes (this choice was also supported by subsequent analyses).

The results of Raman spectroscopy (Figure 2B) show that the increase in annealing temperature leads to a shift of the peaks responsible for the W–O–W and O–W–O bonds by approx. 10 cm^–1^. The shift can be attributed to the formation of CdWO_4_ (the bonding energies of O–W–O and W–O–W are different from those in the WO_3_ sample). The spectral region near 275 cm^–1^ (Figure 2C), corresponding to the crystalline structure of CdS, shows a gradual increase in the characteristic CdS peak intensity after annealing up to 700 °C and the disappearance of this peak after annealing at 900 °C (at this temperature, a partial sublimation of CdS occurs). Raw XPS data (Figure 2D and Table S1) reveal that the ratio between Cd and S concentrations changes with increasing annealing temperature. The change may be due to removal during annealing of a part of the sulfur atoms. The positions of the characteristic tungsten peaks (W 4d) show that the peaks shift to a lower energy (Figure 2E), which is especially pronounced after annealing at 700 °C. This phenomenon is accompanied by a widening of the characteristic oxygen peaks (O 1s) (Figure 2E), due to the presence of oxygen species adsorbed at the WO_3–*x*_ defects.^64,65^ The results of the high-resolution XPS spectra fit are presented in Figure S1 (W, Cd, and O characteristic peaks, measured on the WO_3_, CdS, and WC-700 samples). In the case of W, the peaks are shifted toward lower binding energy and an apparent increase of the W^5+^/W^6+^ ratio is observed, both indicating partial tungsten reduction.^66,67^ In the case of oxygen, an additional peak at 532.8 eV appeared after sample annealing, which should be attributed to oxygen vacancies, mentioned above. No pronounced changes in the position and ratio of the Cd-related XPS peaks were observed, except their shift toward higher binding energy after the introduction of CdS into the Z-scheme design. This shift can be explained by the loss of electrons in contrast to W peaks mentioned before.^66,67^

**Figure 3:**
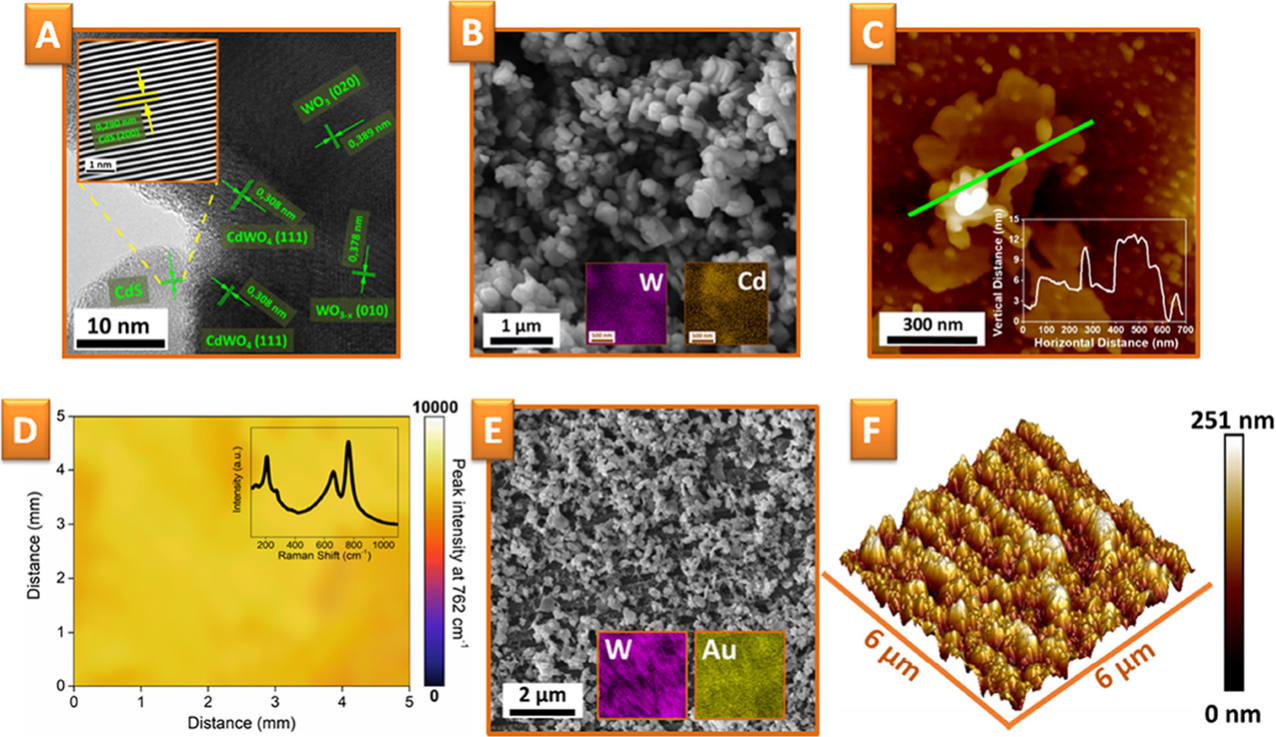
(A) HRTEM image of WO_3_/CdWO_4_/CdS, prepared by annealing at 700 °C of pristine WO_3_/CdS flakes (the inset reveals the FFT image of CdS); (B) SEM images of WC-700 flakes; (C) AFM scan and surface profile of a single WC-700 flake; (D) Raman mapping of the WC-700 distribution on the Au grating surface; and (E) SEM–EDX and (F) AFM measurements of the Au/WC-700 photoelectrode surface after the deposition of WC-700 flakes on Au grating.

The formation of CdWO_4_ is evident from the HRTEM images, from which the interplanar spacing of the initial WO_3_/CdS (Figure S2) and WO_3–*x*_/CdWO_4_/CdS formed after annealing can also be analyzed (Figure 3A). HRTEM images taken from different areas of WO_3–*x*_/CdWO_4_/CdS flakes reveal the presence of several interplanar spacing values, characteristic for WO_3–*x*_ (010), CdS (200), and more importantly, CdWO_4_ (111),^68^ which was not visible in pristine WO_3_/CdS (Figure S2). Therefore, in this way the annealing-induced formation of the CdWO_4_ phase is definitively confirmed. Furthermore, its interplanar spacing of 0.308 nm (111) is an average of those for WO_3_ (0.378 nm (010)) and CdS (0.290 nm (200)). By the presence of CdWO_4_, the crystal mismatch between WO_3_ and CdS is partially compensated, while the transport of charge carriers and hindering of reverse electron–hole recombination are facilitated.

We also estimated the morphology of WO_3–*x*_/CdWO_4_/CdS. Determined from the SEM images, the lateral size of the WC-700 nanostructures (Figure 3B) in the 300–700 nm range is in good agreement with the TEM (Figure S3) and atomic force microscopy (AFM) (Figure 3C) results. AFM measurements performed on the flakes deposited on the Si substrate show that the thickness of the flakes is approximately 12 nm (Figure 3C). Comparison with WC-0 flakes (see Figures S4 and S5) shows that annealing changes the flake morphology: the flakes become thicker, while their lateral size decreases. Such morphology changes may be beneficial for flake combination with the plasmon-active grating, since the smaller flakes could better penetrate in the grating valleys and a complete conformal surface coverage could be achieved in an easy manner (pristine grating morphology and profile are presented in Figure S6). Finally, we performed BET measurements (Figure S7), which indicate that the surface area of WC-700 powder is about 12.2 ± 0.3 m^2^/g and the pore volume is 0.013 ± 0.002 cm^3^/g (the smaller surface area can be explained by the tendency of the 2D flakes to stick together, which prevents the penetration of nitrogen molecules into the space between them).

### 2.3 Coupling of WO_3–*x*_/CdWO_4_/CdS with the Plasmon-Active Grating Surface

Based on the previous results, we used the WC-700 samples for coupling the flakes with the Au grating surface (annealing at 700 °C leads to the formation of CdWO_4_ without the material degradation observed at higher temperatures). For immobilization of WC-700 flakes on the surface of the grating, we used the technique optimized in previous works.^12,47^ The homogeneity of the grating surface coverage, which is a key factor in the creation of an effective artificial leaf structure, was checked by Raman mapping (in particular, surface-enhanced Raman spectroscopy (SERS)). The SERS spectrum of WC-700 reveals the vibrational bands of WO_3_, located at 762 cm^–1^ (W–OW), 652 cm^–1^ (W_2_O_6_/W_3_O_8_), 210 cm^–1^ (O–W–O), and 136 cm^–1^ (lattice vibrations) (Figure 3D inset). The SERS mapping (Figure 3D), performed using the most pronounced 762 cm^–1^ band, shows relatively homogeneous coverage of the grating surface, with a resolution limit determined by the excitation beam spot of ca. 30 μm^2^. SEM–EDX measurements (Figure 3E), performed at higher magnification, also indicate the homogeneous coverage of the grating surface, evident from both the morphology changes and the EDX mapping. Finally, the AFM results also confirm a change in local grating morphology (Figure 3F vs Figure S6) but also preservation of overall periodicity.

### 2.4 Water Splitting in the Photoelectrochemical Regime

**Figure 4:**
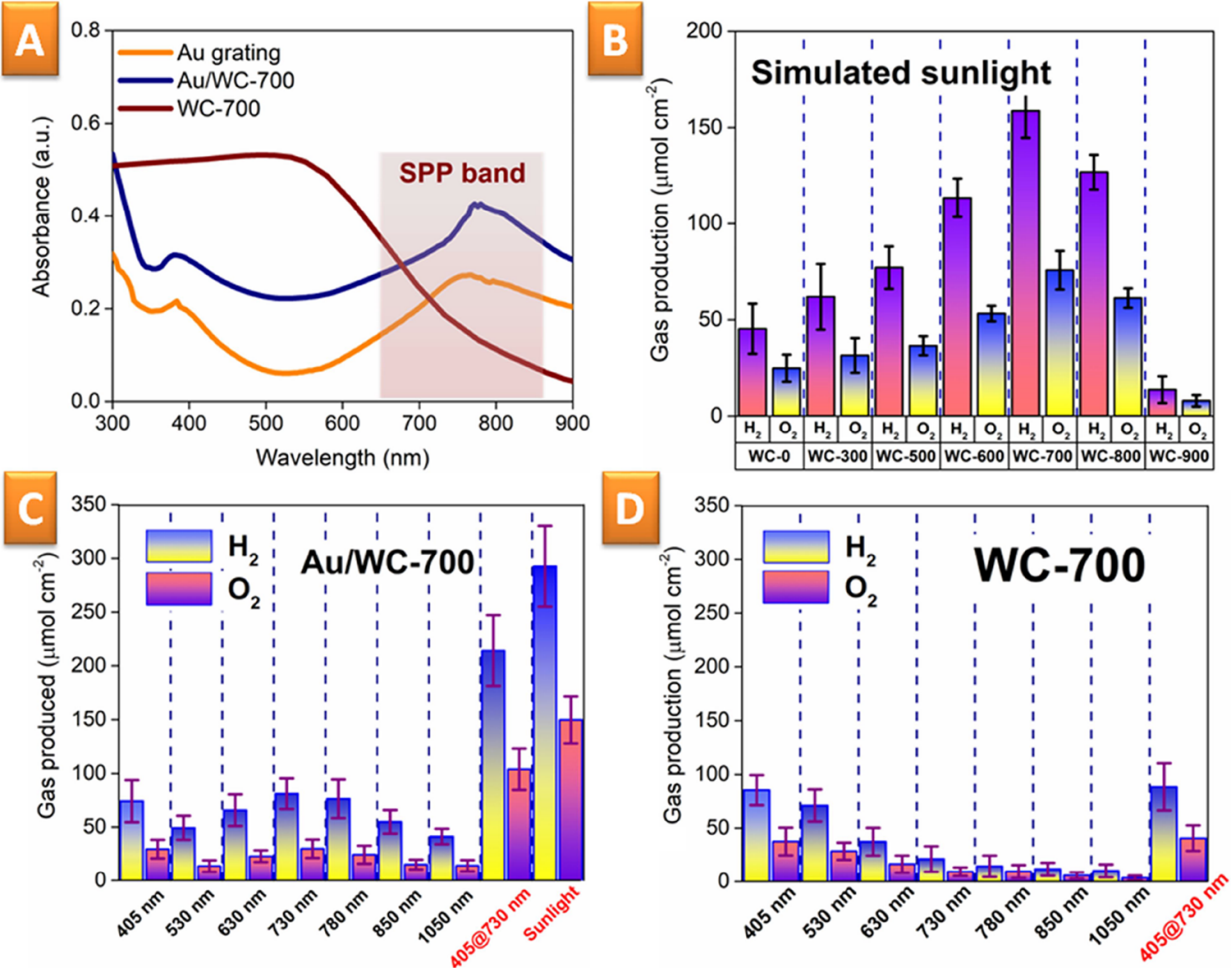
(A) UV–vis absorption spectra of WC-700 flakes, pristine Au grating, and Au grating with deposited WC-700; (B) amounts of hydrogen and oxygen on the Au/WC–X surface as a function of pristine WO_3_/CdS annealing temperature (results are given only for solely sunlight-induced water splitting); (C) amounts of hydrogen and oxygen produced on the illuminated Au/WC-700 surface as a function of the light wavelength; and (D) results of comparative measurements—water splitting achieved on the surface of WC-700 flakes not coupled with the plasmon-active substrate.

As mentioned above, the created WO_3–*x*_/CdWO_4_/CdS materials can efficiently participate in water splitting, due to a suitable position of the redox-active band. It should also be noted that the CdWO_4_ phase is located between CdS and WO_3–*x*_ and therefore cannot participate in water splitting and acts solely as a promoter of charge separation. Water-splitting half-reactions can proceed on the CdS surface (S sites or anion defects, according to refs (69, 70), which is catalytically active in the hydrogen evolution reaction (HER) and on the WO_3–*x*_ surface (top of the W atom, edges of flakes or oxygen vacancies, according to refs (71−73)), which is catalytically active in the oxygen evolution reaction (OER). The functionality of the created WO_3–*x*_/CdWO_4_/CdS flakes, deposited on the plasmon-active Au grating, was demonstrated in two distinct modes: (i) with the utilization of photoelectrochemical water splitting and (ii) with only sunlight-induced water splitting. The UV–vis spectra of the Au grating with deposited flakes are presented in Figure 4A. Comparison with separate materials (Figure S8) indicates that the created hybrid structure can efficiently absorb the photons of visible light, those close to the UV wavelengths (which is ensured by flakes), and those at longer wavelengths (due to surface plasmon polariton (SPP) excitation).

### 2.5 Utilization of Photoelectrochemical Water Splitting

First, we estimate the impact of annealing temperature on the electrochemical activity of the samples (WC–X flakes deposited on Au grating). The linear sweep voltammetry (LSV) of both water-splitting half-reactions (HER and OER) reveals the slight shift of LSV curves as a function of annealing temperature (Figure S9) and more pronounced shifts under illumination with simulated sunlight (Figure S10). In the last case, the more pronounced shifts of LSV curves were observed for WC-700, in agreement with the above material characterization and CdWO_4_ phase formation. We also examined the role of individual light wavelengths on the increase in PEC activity in HER and OER (Figure S10C,D). It was found that the PEC activity is almost equally contributed by both the photons with higher energy (i.e., illumination at 405 nm light wavelengths), absorbed by the WC-700 flakes, and the photons with lower energy (i.e., illumination at 730 nm light wavelengths), responsible for SPP excitation. Moreover, the impact of simultaneous illumination with double wavelengths or simulated sunlight illumination also leads to a pronounced decrease of overpotentials in both HER and OER processes. Finally, electrochemical impedance spectroscopy (EIS) results also indicate the apparent decrease of interface charge transfer resistance, achieved through Z-scheme annealing and double wavelength illumination, as is evident from the apparent decrease of characteristic EIS semicircles (Figure S11).

### 2.6 Solely Sunlight-Induced Water Splitting

In the next step, solely sunlight-induced water splitting was performed. Like in the PEC case, we estimated the impact of different temperatures and wavelengths on the amounts of produced hydrogen and oxygen using the simulated sunlight as the sole energy input (Figure 4B,C). Almost all samples were able to produce hydrogen under simulated sunlight. However, the highest amount of produced hydrogen was observed in the case of WC-700 samples, which overcome the unannealed WC-0 samples 3.5 times. Moreover, we also observed closer to stoichiometric amounts of hydrogen and oxygen in the case of WC-700 (unlike the WC-0 samples, where the holes excited by sunlight are partially consumed in S^2–^ oxidation, leading to gradual material degradation^74,75^).

The impact of individual light wavelengths in the case of more efficient WO_3_ is presented in Figure 4C. The obtained results indicate that the created structures can produce hydrogen under illumination with almost all wavelengths of the sunlight spectrum. It can be assumed that both the water-splitting half-reactions proceed under light triggering (shorter wavelength) and plasmon triggering of the WC-700 structure, both leading to the creation and separation of redox-active electrons and holes. More interesting results were observed with the utilization of double wavelength illumination. One wavelength (405 nm) is directly absorbed by WC-700, leading to generation of electron–hole pairs. The longer wavelength (730 nm) is converted to a surface plasmon, which can also generate electrons and holes in WC-700 or separately accelerate existing ones. In the case of double wavelength illumination, we observed the synergistic increase in the amounts of hydrogen and oxygen, which exceed the sum of the gas amounts produced under separate illumination at 405 nm (2.84 times) and 730 nm (2.63 times) wavelengths. Similar results were observed for water splitting with a simulated sunlight simulator: the amount of hydrogen reached 291.7 μmol cm^–2^ (with the stoichiometric amount of oxygen). For comparison, the results obtained with WC-700 flakes deposited on the nonplasmonic support (glassy carbon electrode) are shown in Figure 4D. In this case, the utilization of shorter wavelengths leads to the production of similar amounts of both gases. However, illumination of WC-700 flakes, deposited on nonplasmonic surfaces, with longer wavelengths did not produce any hydrogen or oxygen, thereby confirming the key role of plasmon triggering.

### 2.7 Comparison of the Efficiency of the Proposed Structure with Previous Results

In our previous work, we have already shown the possibility of creating an artificial leaf structure with a simple Z-scheme deposited on a plasmon-active surface. With such a system, we produced 173 μmol cm^–2^ of hydrogen. In this work, we significantly surpassed this result thanks to the formation of the more advanced Z-scheme design. The previously proposed synergy in double wavelength triggering of the Z-scheme coupled with the plasmon-active substrate supposes the excitation of electron–hole pairs. The electrons and holes are separated and/or accelerated under the influence of the electrical component of the plasmon wave, and in this way, they receive additional energy. After charge separation, the electrons should pass through the interphase between the WO_3_ and CdS semiconductors. The formation of the CdWO_4_ interphase greatly facilitates this process and makes possible a more efficient water splitting. Another favorable process could be the formation of oxygen vacancies in WO_3–*x*_ structures, which can positively affect oxygen evolution^76,77^ (the reaction is commonly considered as a limiting step).

We also compared our approach with those of other groups working on artificial leaf (or Z-scheme-based water splitting) research (see Table 1(70,78−90)). As can be seen, our results are comparable with the best results of other authors. In particular, most of the published works utilize the STH (solar-to-hydrogen) value as a quantitative parameter of light-induced water splitting efficiency. We recalculated our results for STH and obtained a value of 0.64% (and the corresponding high amount of produced hydrogen—Table 1), which significantly exceeds some previously published ones.^78−82,87,89^ Our STH values (or the amounts of hydrogen produced) are somewhat lower than the best ones, which, unlike us, were obtained with sacrificial agents.^70,83,84,88,90^ Finally, the quantum efficiency (QE) of hydrogen production was calculated for the more interesting case of the utilization of double wavelength triggering of WC-700 flakes deposited on the plasmon-active grating surface. Using the known light irradiance on the sample surface and the amount of hydrogen produced, we obtain QE = 79.33%, which is close to those recently reported in recent top papers.^91−93^ Therefore, we can conclude that from the point of effectiveness and efficiency, the proposed approach is fully competitive, especially for direct water splitting without the addition of sacrificial agents, commonly used and consumed during water photolysis.

**Table 1:** Comparison of Our Results with Previously Published Ones on Artificial-Leaf-Based Water Splitting

photocatalytic system	cocatalyst	sacrificial agent	gas amount (μmol g^–1^ h^–1^)	STH (%)	ref
CdS@NiO	Ni	TEOA	O_2_: 20.1	0.00021	(78)
			H_2_: 42.5		
2D/2D Ti_3_C_2_/g-C_3_N_4_	3 wt % Pt	TEOA	H_2_: 72.3	0.072	(79)
P-doped Zn_0.5_Cd_0.5_S_1–*x*_/Bi_4_NbO_8_Cl	no	no	H_2_: 13.85	0.15	(80)
			O_2_: 6.89		
ZnCdS/Co-MoS_*x*_	no	lactic acid	H_2_: 8.75	1.4	(81)
Zn_*x*_Cd_1 – *x*_In_2_S_4_/g-C_3_N_4_	no	TEOA	H_2_: 3.41	2.4	(82)
SrTiO_3_/Bi_4_Ti_3_O_12_	no	methanol	H_2_: 1265	0.19	(83)
Rh_0.5_Cr_1.5_O_3_-loaded AgTaO_3_	no	no	H_2_: 400	0.13	(84)
			O_2_: 192		
Te/SnS_2_	Ag	no	H_2_: 332.4	0.5	(85)
			O_2_: 166.2		
LaFeO_3_-g-C_3_N_4_-BiFeO_3_	1 wt % Au	methanol	H_2_: 698.4		(86)
g-C_3_N_4_/ITO/Co-BiVO_4_	Co	no	H_2_: 95.41	0.028	(87)
			O_2_: 40.23		
ZnS/ZnO	no	Na_2_S/Na_2_SO_3_	H_2_: 95.41		(88)
			O_2_: 40.23		
BaTaO_2_N/BiVO_4_	Na-Pt, Cr_2_O_3_, Zr, CoO_*x*_, Au	methanol	H_2_: ≈5000	0.022	(89)
WO_3_/BP/g-C_3_N_4_	no	no	H_2_: 400		(70)
TiO_2_-graphene-Ta_3_N_5_	1 wt % Pt	no	H_2_: 180		(90)
Au grating/WO_3–*x*_/CdWO_4_/CdS	no	no	H_2_: 1173.12	0.64	this work
			O_2_: 602.27		

### 2.8 Stability and Photodegradation

**Figure 5:**
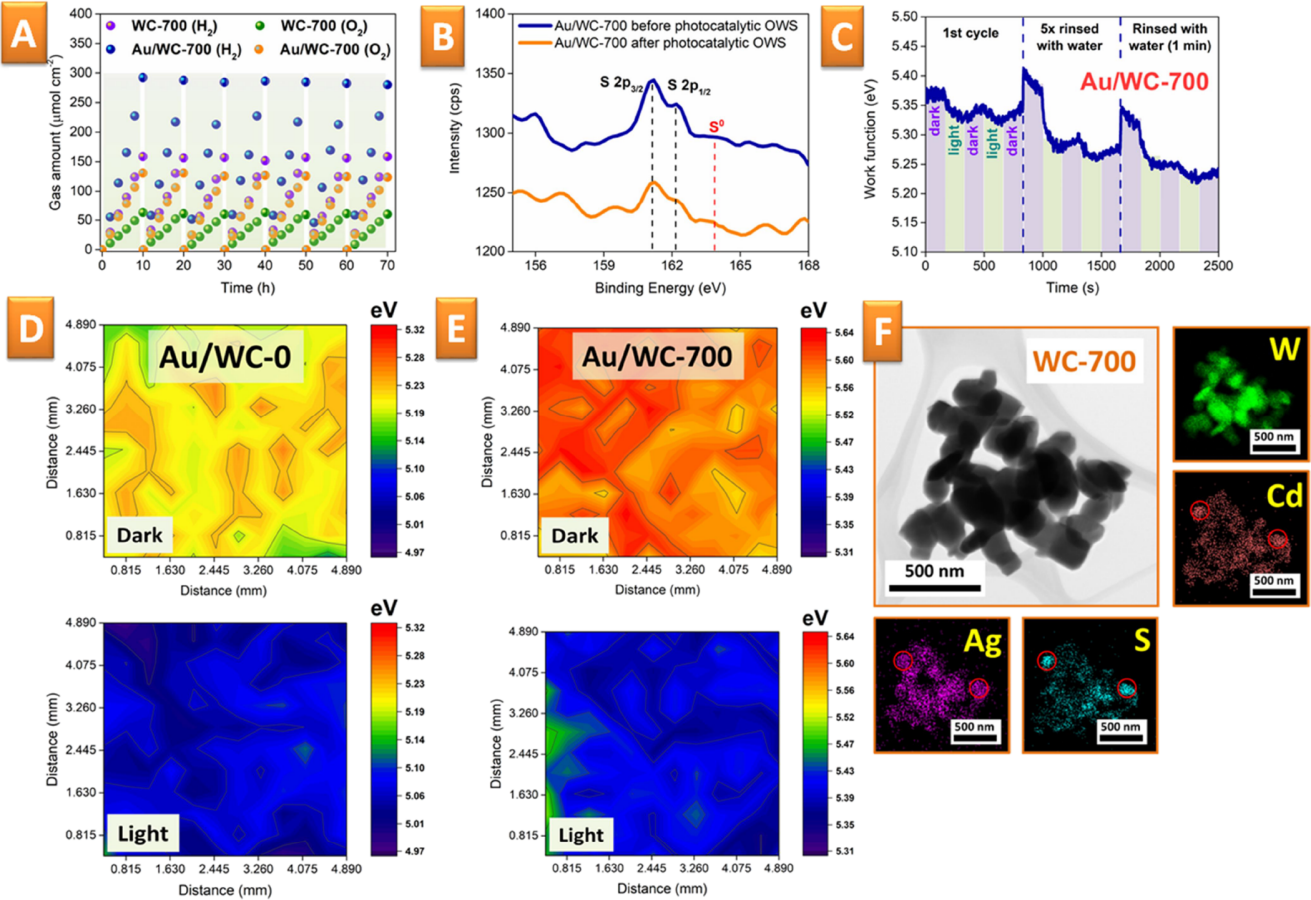
(A) Several subsequent cycles of light-induced water splitting on the Au/WC-700 surface with a total duration of 70 h; (B) details of the S 2p XPS peak of WC-700 measured before and after use in water splitting; (C) determination of the WF on Au/WC-700 in darkness, under illumination, and after rinsing of the samples with water; (D,E) Kelvin probe mapping of Au/WC-0 and Au/WC-700 performed in darkness and under simulated sunlight illumination; and (F) HRTEM-EDX measurements of WC-700 flakes after their illumination in Ag^+^ solution.

An important problem emerging in the design of the Z-scheme is photodegradation. Especially in the case of materials coupled with sulfides, an insufficient transfer of photoexcited holes from one semiconductor to another can induce sulfur oxidation (i.e., formation of S^0^) leading to significant loss of catalytic activity.^68^ We observed this phenomenon in the case of Au/WC-0 samples, and our result on nonstoichiometry of the hydrogen and oxygen (Figure 4B) correlates well with the previously reported ones.^94,95^ In fact, the XPS analysis, performed after water splitting, confirmed sulfur oxidation (Figure S12). However, the preliminary annealing of the samples resulted in almost stoichiometric amounts of oxygen and hydrogen, which were kept during several subsequent cycles of water splitting with a total duration of 70 h (Figure 5A). Therefore, we concluded that the created interphase of CdWO_4_ can facilitate charge transport between CdS (responsible for HER) and WO_3_ (responsible for OER) and can make sulfur oxidation less probable. The preservation of sulfur was additionally confirmed by XPS analysis of the samples performed after the water-splitting experiments (Figure 5B vs Figure S12).

### 2.9 Confirmation of the Z-Scheme Mechanism

The mechanism of Z-scheme action is based on the separation of charge carriers between coupled semiconductors. To confirm this assumption, we performed a range of additional experiments, using the scanning Kelvin probe (SKP), with the aim to determine the light-induced changes of the surface work function (WF) that can reflect the charge spatial separation^96^ (a more detailed and specific description is given in the Supporting Information (SI)). In particular, we used the SKP measurements for the identification of the surface of charge generation on the samples under sunlight irradiation. In our setup, the difference in the WF corresponds to photovoltage generation on the sample surface,^97^ while lower WF values correspond to positive photovoltage. As shown in Figure 5C, the Au grating/WC-700 samples show an intense response to light illumination with a photovoltage of up to +100 mV. For comparison, Figure S13 shows the initial Au grating substrate response, which is significantly lower compared to that of Au grating/WC-700. Thus, we can conclude that the measured photovoltage does not come from the grating itself but from the holes transferred to the Au grating/WC-700 sample surface. After the illumination is switched off, the WF tends to return to its initial value, indicating the gradual recombination of the excited/separated electron and holes. The full return is very slow (overnights), indicating that the holes are retained at the surface even when the illumination is off. This finding explains why the response of the WF to repeated illumination quickly diminishes. However, exposing the Au/WC-700 sample to water, even briefly for a few seconds during five dips, leads to an immediate return of the WF to its “dark” state and recovery of the pronounced photovoltage response. Thus, the recovery can be attributed to extinction of light-induced charge carriers, which are consumed in water splitting. The WF of Au/WC-700 remains relatively stable (within 0.05 eV) during several irradiation cycles even after water exposure, which corroborates the absence of material photoinduced or chemically induced degradation.

We also performed the SKP mapping (Figures 5D,E) to assess WF homogeneity and the effect of photovoltage across the Au grating/WC-0 and Au grating/WC-700 sample surface. Both the dark and light SKP maps exhibit some large-scale features, but the WF variations across the samples are within only 0.05 eV so that the WF distribution can be considered as a homogeneous one. In darkness, the WFs for the Au/WC-0 and Au/WC-700 samples differ by about 0.4 eV, the difference reflecting their different compositions and structures. The calculated photovoltage (as a light–dark difference) also shows some inhomogeneities. On average, it is +0.2 and +0.3 eV on the Au/WC-0 and Au/WC-700 surfaces, respectively. This indicates that there are more free charge carriers (holes) on the surface Au/WC-700, probably due to better charge separation and less pronounced dissipation on the semiconductor interface. Interestingly, the irradiation of Au/WC-0 after rinsing with water results in a significant increase in the material WF, indicating a smaller number of electrons in the conduction band of CdS, which, however, could be increased by oxidation of CdS under light irradiation.

To verify charge separation between semiconductors and the fact that HER and OER proceed in spatially separated places, we performed additional experiments using Ag^+^ ions’ oxidation and the formation of an insoluble Ag^0^ phase on the WC-700 surface. The results of the EDX mapping of WC-700 flakes after illumination with simulated sunlight in the presence of Ag^+^ ions are given in Figure 5F. The mapping clearly shows that the spatial distribution of Ag overlaps well with the signal from Cd and S (regions marked by red circles in Figure 5F), while the Ag atoms are missing in place of W signal appearance. Therefore, we can conclude that the reduction process (Ag^+^ → Ag^0^ as an analogy of 2H^+^ → H_2_) takes place solely on the surface of one semiconductor, confirming in this way the general mechanism of the Z-scheme action.

An improvement in the efficiency of the Z-scheme after the annealing-induced formation of the CdWO_4_ interface and the appearance of defects in the tungsten oxide structure was additionally confirmed by photoluminescence (PL) spectroscopy with utilization of a luminescence probe (coumarin). The reaction of coumarin with OH· radicals (created from OH^–^ chemical groups under their interaction with photoexcited holes on the surface of WC-700) leads to the formation of 7-hydroxycoumarin, which is a highly luminescent compound.^58^ Subsequently, the measured PL spectra are presented in Figure S14A. As is evident, the initial coumarin solution had no luminescence. Its interaction with photoexcited WC-700 flakes leads to 7-hydroxycoumarin formation and appearance of luminescence, whose intensity increases with time. Figure S14B shows the time-dependent luminescence increase as a function of the semiconductor used (separately prepared CdS nanostructures and WO_3_, WC-0, and WC-700 flakes were used). In the case of CdS, no luminescence was observed, indicating the absence of radical formation (i.e., absence of holes with appropriate redox activity), as could be expected from the position of the CdS valence band. The use of WO_3_, instead of CdS, led to the appearance of luminescence, indicating the presence of redox-active holes in the system, which can participate in the oxidation of coumarin or in water-splitting half-reaction. The coupling of WO_3_ with CdS (i.e., in the case of WC-0 sample utilization) significantly increases the reaction efficiency, as indicated by a more pronounced luminescence increase (Figure S14B). Preliminary annealing of WC-0 (preparation of WC-700 samples) leads to more pronounced luminescence increase and even a greater number of redox-active holes. Therefore, we can conclude that the combination of CdS and WO_3_ significantly increases the number of redox-active charge carriers capable of participating in the half-reaction of water splitting (oxygen evolution). Preliminary annealing, with the formation of the CdWO_4_ interface and the appearance of vacancies in the structure of tungsten oxide, further increases the number of redox-active holes, thus increasing the photocatalytic activity of the formed flakes.

**Figure 6:**
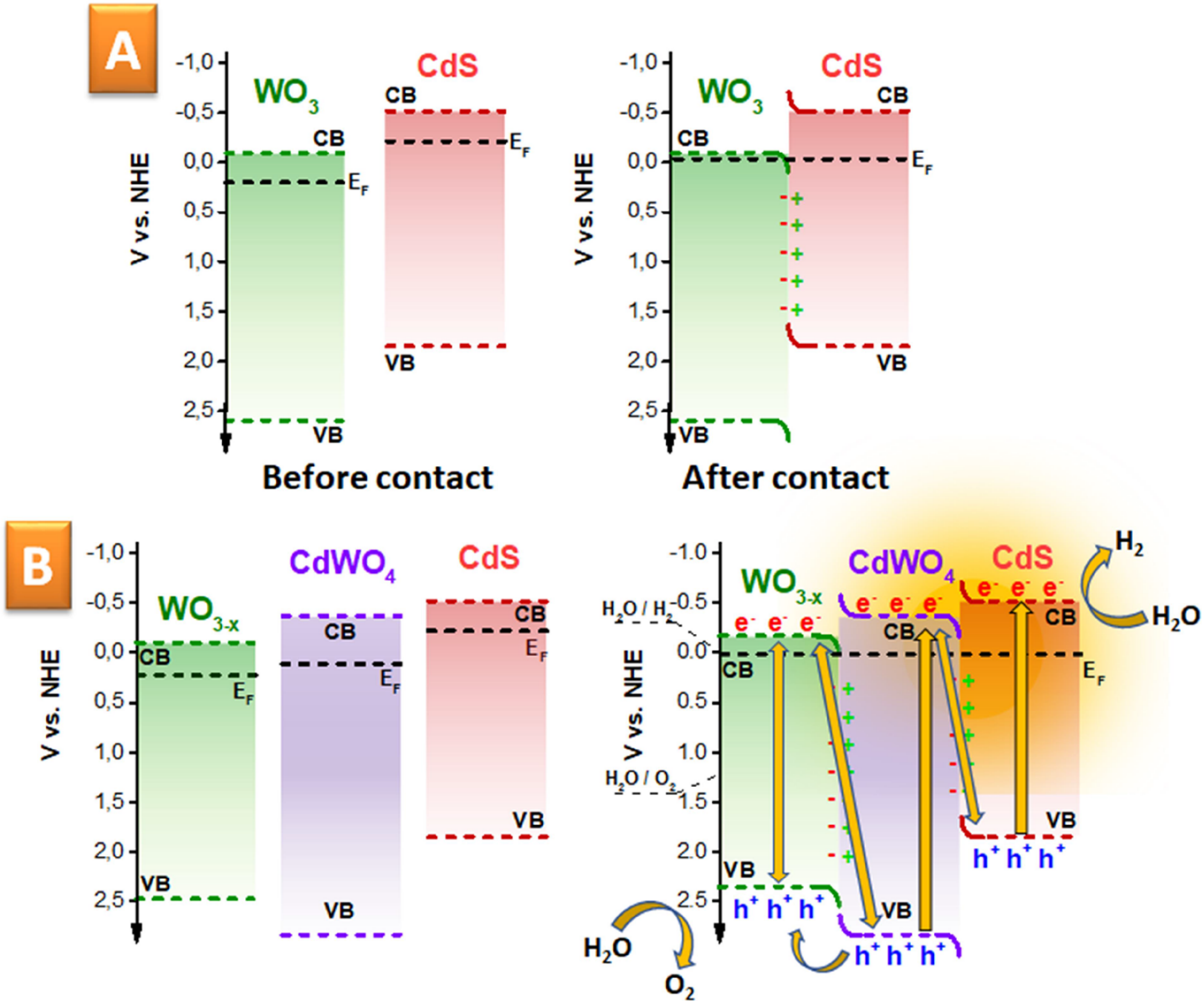
Proposed band position and alignment in the direct Z-scheme(s) and water splitting: (A) simple case of the Z-scheme (coupled CdS and WO_3_) and (B) more sophisticated case of the coupled WO_3–*x*_/CdWO_4_/CdS structure.

We also estimated the position and alignment of the CB and VB, which occurred due to the coupling of the CdS and WO_3_ flakes and subsequent annealing during Z-scheme creation and utilization (Figure 6). In the case of the WC-0 structure (i.e., coupled WO_3_-CdS before annealing), it was a relatively simple task, since the positions of the CB and VB as well as the Fermi level of both semiconductors can be easily determined using Tauc and Mott–Schottky plots (Figure S15A–D) in combination with the low binding energy part of the XPS plot. The results of the coupling of WO_3_ and CdS are presented schematically, where the “right” opposition of CB and VB, as well as their alignment, indicate the creation of a direct Z-scheme (Figure 6A), which supports the recombination of residual holes from CdS and residual electrons from WO_3._^98−100^ However, in the case of the present WO_3–*x*_/CdWO_4_/CdS structure, created by Z-scheme annealing, the direct investigation of the position of the VB and CB is complicated by the fact that WO_3–*x*_ and CdWO_4_ materials are created directly during annealing and cannot be subsequently separated (and measured) from each other. Thus, we were forced to utilize the available literature data for WO_3–*x*_ and CdWO_4._ Therefore, the proposed band position for WO_3–*x*_, CdWO_4_, and CdS is presented in Figure 6B. Based on this result, we can also propose the band alignment after semiconductor coupling and photon (or plasmon)-induced water splitting (control XPS measurements indicate a value of 2.81 eV as the difference between *E*_F_ and the VB via the intersection method, close to the predicted one—Figure S15E). We suppose that electrons can be excited under plasmon triggering or photon absorption in the case of all materials, including WO_3–*x*_, CdWO_4_, and CdS. However, since CdWO_4_ is sandwiched between CdS and WO_3–*x*_, it cannot directly participate in the water splitting. On the other hand, the holes from CdWO_4_ can be injected into WO_3–*x*_, which has a suitable redox position of the CB and abundant catalytic places for oxygen evolution. Therefore, water oxidation proceeds on the WO_3–*x*_ side, with the consumption of photo- (or plasmon) excited holes from this material or holes injected from CdWO_4_. Hydrogen evolution occurs on the CdS side, with the utilization of photo- (or plasmon) excited electrons. The residual holes from CdS recombine with the residual electrons from the CdWO_4_ VB. Finally, the residual electrons from WO_3–*x*_ can recombine with “inner” holes (undesired process, partially compensated by external hole injection) or with holes from CdWO_4_ (desired process).

## 3 Conclusions

In this work, we demonstrated the creation of an advanced Z-scheme through the annealing of initially prepared and coupled WO_3_ and CdS semiconductors. The annealing process results in the creation of an additional CdWO_4_ phase and the production of excessive oxygen vacancies in the initial stoichiometric WO_3_ (i.e., creation of more active holes in the OER WO_3–*x*_ phase). The presence of CdWO_4_ between WO_3–*x*_ and CdS facilitates charge transfer between semiconductors and compensates for the lattice mismatch between them, both leading to a significant improvement in the Z-scheme efficiency. The annealed Z-scheme was subsequently coupled with a plasmon-active gold grating to create an artificial leaf design. After the optimization of the annealing procedure and deposition on the gold grating, the created structure was used for water splitting with solely sunlight energy input. Effective water splitting was achieved without commonly used sacrificial agents and proceeds with high efficiency (H_2_: 29.4 μmol/cm^2^/h and O_2_: 15.1 μmol/cm^2^/h). The stoichiometric amounts of hydrogen and oxygen are produced under sample illumination, while photodegradation (in particular, sulfur oxidation) is significantly suppressed by the presence of the CdWO_4_ phase. Finally, we performed a range of control experiments, indicating the spatially selective HER and OER processes proceeding in the proposed artificial leaf design, as well as the creation of separated charge carriers (under illumination in air) and their immediate consumption after the samples come into contact with water.

## 4 Experimental Section

### 4.1 Used Materials and Sample Preparation

A detailed description of the materials used, sample preparation, and measurement techniques is given in the SI.

### 4.2 Preparation of WC–X on the Active Surface

Briefly, WO_3_ nanoflakes were prepared using the previously reported route using hydrothermal synthesis and subsequent annealing at 400 °C for 10 h. The deposition of CdS on the surface of WO_3_ proceeded through immobilization of cadmium cations on the surface of WO_3_ and subsequent Na_2_S addition.^8,60^ The WO_3_-CdS flakes purified by several cycles of centrifugation/washing/redispersion were subjected to annealing in a nitrogen atmosphere at different temperatures for 4 h. The created flakes were deposited on a plasmon-active Au grating prepared by Au sputtering on a periodically patterned polycarbonate surface. The uniform distribution of the flakes on the Au grating surface was achieved by optimization of the deposition method and conditions (slow-rate spin coating was used, and deposition was performed from methanol suspension).

### 4.3 Photocatalytic Water-Splitting Test

In only light-induced water-splitting experiments, samples with a surface area of 3 × 3 cm^2^ were immersed in a self-made reaction cell and illuminated with simulated sunlight (Solar Simulator SciSun-300, Class AAA). The intensity of light on the sample surface was adjusted to be close to the common intensity of sunlight (100 mW/cm^2^). The amount of H_2_ and O_2_ evolved was determined at 2 h intervals using an online gas chromatography system (GC-7920).

## Supporting Information Available

Detailed description of the materials used, sample preparation, and measurement techniques; XPS measured element concentration (in at %) on the surface of WO_3_/CdS flakes annealed at different temperatures; SEM, HRTEM, AFM, and TEM images of the pristine WO_3_/CdS flake; fittings of high-resolution XPS spectra; N_2_ adsorption/desorption isotherms; AFM image of the pristine Au grating and its surface profile; UV–vis absorption spectra of Au grating and WC–X flakes; results of water splitting performed in the photoelectrochemical mode: LSV curves in HER and OER potentials, measured under illumination and in the dark; EIS spectra; XPS-based demonstration of illumination-induced sulfur oxidation in the case of pristine WO_3_/CdS flakes; PL experiments; WF measured on the Au grating surface as a function of sample illumination or rinsing with water; and estimation of VB and CB positions and alignments, Mott–Schottky and Tauc plots (PDF)
