# Supplementary material for: Creation and Plasmon-Assisted Photosensitization of Annealed Z-Schemes for Sunlight-Only Water Splitting
Source: ACS Appl Mater Interfaces. 2023 Jun 6;15(24):29072–83. doi: 10.1021/acsami.3c02884 (PMC10288442; doi:10.1021/acsami.3c02884)
Supplement: Supplementary file 1 — am3c02884_si_001.pdf [file am3c02884_si_001.pdf]

Supporting Information for:

Creation and Plasmon-Assisted Photosensitization of Dual Z-Scheme for Sunlight-Only Water Splitting

Denis Zabelin[a], Kamil Severa[a], Jaroslav Kuliček[b], Bohuslav Rezek[b], Anastasiia Tulupova[a], Roman Elashnikov[a], Anna Zabelina[a], Vasilii Burtsev[a], Petr Sajdl[a], Elena Miliutina[a], Vaclav Svorcik[a], Oleksiy Lyutakov[a]*

[a] *Department of Solid State Engineering, University of Chemistry and Technology, 16628 Prague, Czech Republic*

[b] *Faculty of Electrical Engineering, Czech Technical University in Prague, 16627 Prague, Czech Republic*

---

* *Corresponding author: lyutakoo@vscht.cz*

1. Experimental

1.1. Materials

Sodium tungstate dihydrate (≥ 99 %), tetrafluoroboric acid (48 wt. % in the water), cadmium chloride monohydrate (99.995 %), sodium sulphide nonahydrate (≥ 99.99 %) and deionized water were purchased from Sigma-Aldrich. Ethanol (p.a. 96 %) and methanol (p.a. 99.96 %) were purchased from Lachner. DVD-R disks were purchased from Verbatim (USA). Au target was purchased from Safina (purity of 99.99 %, Czech Republic)

1.2. Sample preparation

1.2.1. Preparation of WO_3 nanoflakes. WO_3 nanoflakes were prepared using previously reported route [S1]. Briefly, tetrafluoroboric acid solution was added dropwise into sodium tungstate water solution under vigorous stirring. Obtained H_2WO_4 suspension was transferred to a Teflon autoclave, which was placed in an oven and heated at 180 °C for 10 hours. The resulting $WO_3 \cdot H_2O$ was collected by centrifugation and washed twice with deionized water and ethanol and then dried in oven at 60 °C for 5 hours. The resulting powder was subjected to calcination at 400 °C for 3 hours to obtain WO_3 nanoflakes powder.

1.2.2. Preparation of WO_3-CdS photocatalyst system. Deposition of CdS on the surface of WO_3 was performed in two subsequent stages: first, cadmium cations were immobilized on WO_3

surface by adding $CdCl_2$ solution to the WO_3 aqueous suspension under vigorous stirring for 30 min, and then a Na_2S aqueous solution was added with the suspension getting a yellowish tint [S1, S2]. The resulting water-soluble sodium chloride was removed from the product by centrifugation and washed twice with deionized water. The substance was further dried in an oven at 60 °C for 2 hours.

1.2.3. Preparation of WO_3/$CdWO_4$/CdS photocatalyst. The previously obtained WO_3-CdS system was placed in a quartz crucible and then subjected to annealing in nitrogen atmosphere at 300, 500, 600, 700, 800, or 900 °C for 4 hours. The samples were further referred as WC-X, where X is the annealing temperature.

1.2.4. Preparation of plasmon active Au grating. The plasmon-active periodical Au grating was prepared by separating two transverse portions of the DVD-R disc (periodical polycarbonate template was obtained in this way) and subsequent sputtering of 30 nm thick Au film on polycarbonate surface (magnetron sputtering, 40 mA, 300 s).

1.2.5. Deposition of WC-X on grating surface. The uniform distribution of WC-X flakes on Au grating surface was achieved by spin-coating method. Briefly, the optimal deposition parameters were: WO_3/$CdWO_4$/CdS concentration in methanol suspension of 1.1 mg mL^{-1}, the speed rotation 250 rpm and time of spin-coating 2 min.

1.3. Measurements techniques

1.3.1. Materials characterization. X-ray photoelectron spectroscopy (XPS) was performed using an Omicron Nanotechnology ESCAProbeP spectrometer with a monochromated Al K Alpha X-ray source operating at 1486.6 eV. The energy resolution was 0.4 eV for the survey study and 0.1 eV for the high-resolution XPS spectra measurements. Raman spectra were measured using ProRaman-L spectrometer (Enwave Optronics) (Laser power 90 mW) with 785 nm excitation wavelengths. Spectra were measured 30 times, each of them with 3 s accumulation time. UV-Vis absorption spectra were obtained on a Lambda 25 UV/Vis/NIR Spectrometer (PerkinElmer, USA). X-ray diffraction microscopy spectra were recorded on a microXRD D8 Discover diffractometer for 30 min using Cu Kα radiation (1.5405 Å) 3 at 30 mA and 40 kV. HRTEM measurements were performed using an EFTEM Jeol 2200 FS microscope (Jeol, Japan). SEM-EDX images were obtained on LYRA3 GMU (Tescan, CR) equipment using accelerating voltages 10 kV and a beam current of 600 pA. The atomic force microscopy (AFM) surface topographies were measured using the Icon system (Bruker) in Peakforce mode.

1.3.2. Photocatalytic water splitting test. In photo-electrochemical experiments catalytic activities of prepared samples toward HER and OER were studied by linear sweep voltammetry, impedance spectroscopy and chronoamperometry. All electrochemical measurements were

performed under illumination with LEDs (at 405, 530, 630, 730, 780, 850 and 1050 nm wavelengths, power on surface was adjusted to a constant level of 25 mW/cm^2) or in darkness with portable potentiostat PalmSens 4 (Palm Instruments, Netherlands) in a three-electrode cell. The sample was used as a working electrode, Pt as the counter electrode and a saturated Hg/HgO as the reference electrode. Measurements of HER and OER were carried out in N_2-saturated 1M KOH solutions. Electrochemical impedance spectroscopy (EIS) was performed in 1 M KOH solution with the frequency range of 1–100 000 Hz (applied potential was 1.8 V vs RHE). In impedance measurements an equivalent electrical circuit, comprising solution resistance, contact interface resistance, and constant phase element was used.

In solely light induced OWS experiments, photocatalyst was deposited on the surface of periodic gold grating (3 × 3 cm^2) by spin-coating method and then was immersed in self-made reaction cell filled with pure water without any sacrificial agents and illuminated with simulated sunlight (Solar Simulator SciSun-300, Class AAA). The intensity of light on sample surface was adjusted to be closer to common sunlight intensity (100 mW/cm^2). The evolved H_2 and O_2 were determined at 2 h intervals using an online gas chromatography system (GC-7920).

1.3.3. Kelvin Probe measurements and surface potential mapping. Contact potential difference (CPD) was measured by the Scanning Kelvin Probe method (SKP) (KP Technology, U.K.). The CPD data were recalculated to the work function (WF) values using the equation:

$$WF_{sample} = (CPD_{sample} - CPD_{ref}) \times 10^{-3} + WF_{ref} \qquad (1)$$

As the WF reference (WF_{ref}), an Au reference sample from KP Technology was used. SKP measurements were done in a glove box using a grounded steel probe tip of 2 mm diameter. The CPD was measured in the dark-light-dark-light-dark cycle switched on/off the light (simulated sunlight using AM1.5G filter, class AAA solar simulator HAL-C100, Asahi Spectra, Japan), equipped with AM 1.5G filter. In the dark the WF values reflect the intrinsic material properties and possibly retained electrostatic charge too [S3]. Under the illumination, the difference in WF corresponds to photovoltage generation on the sample surface. [S4]. Lower WF (CPD) values correspond in our setup to positive photovoltage, i.e. to generation/transfer of holes on the surface.

Two types of SKP experiments were performed on the samples. First, the CPD was measured as a function of time for 15 min (1250 data points). Multiple locations were tested on each sample. In some particular experiments the samples were let to interact with de-ionized (HPLC quality) water between subsequent cycles of KP measurements. The effect of water was tested briefly (5 dips) or for a longer period of time (1 min submersion). Then the samples were dried by a nitrogen gun and measured by SKP in the dark and under illumination again. In the second type of SKP experiment, mapping of the WF was done across the scanned area of 4.89 × 4.89 mm^2, hence 12 × 12 points

0.4075 mm per point. The sample (rested in dark overnight) was first scanned in the dark and then under illumination immediately again.

1.3.4 Photoluminiscence experiments. The photoluminescent (PL) spectroscopy was used to confirm the heterojunction mechanism and enhanced charge separation. In particular, an aqueous coumarin 1mM solution was used as a probe. Briefly, 0.05 g of WC-700, WC-0, WO_3, or CdS powders were dispersed in coumarin solution and illuminated with simulated sunlight (100 mW cm^{-2}). The PL spectra were measured after different time of illumination, with utilization of 325 nm excitation wavelength (LED lamps, Thorlabs).

1.3.5 Calculation of hydrogen production quantum efficiency. The hydrogen production quantum efficiency **φ** (AQE) can be calculated by following equation:

$$\varphi = \frac{n \cdot N_A \cdot R}{I} \cdot 100\% ,$$

where **n** is the number of involved electrons, $\mathbf{N_A}$ is Avogadro constant, $\mathbf{I = I_1 + I_2}$ is the number of incident photons with 780 nm (I_1) and 405 nm (I_2) wavelengths, **R** is produced H_2 value

For the calculation of incident photons number, the following formula was used:

$$I = \frac{E \cdot t \cdot \lambda \cdot S}{h \cdot c} ,$$

where **E** is the light energy flux density, **t** is irradiation time, **λ** is wavelength, **S** is irradiated area, **h** is Plank's constant, and **c** is speed of light.

For the combination of λ=730 and λ=405 nm we received $I=1.2454 \cdot 10^{19}$ and φ=79.33%

1.3.6. Description of VB and CB calculation (separately prepared WO_3 and CdS)

The levels of VB and CB were determined using the combination of Schottky-Mott and Tauc plots.

The Mott-Schottky plot of semiconductors was plotted according to the following equation (for an n-type and p-type semiconductors) [S6]:

$$\frac{1}{C^2} = \frac{2}{\varepsilon \varepsilon_0 e N_{D(A)}}\left(\pm E \mp E_{fb} - \frac{k_B T}{e}\right);$$

where *C* is the space charge capacitance, *ε* and ε_0 are the dielectric constant of the semiconductor and permittivity in vacuum (8.85×10^{-14} F×cm^{-2}), *e* is the electronic charge, N_D and N_A are the number of donors and acceptors for n-type and p-type semiconductors, respectively, and *E*, E_{fb}, T, and k_B are the applied voltage, flat-band potential, Kelvin temperature, and Boltzmann constant, respectively. According to the slope coefficient of the Mott-Schottky plot (dependence of $1/C^2$ on the potential), the type of semiconductor was determined [S7]. Further, based on the semiconductor type and applied potential, the position of closed band (VB or CB) was determined.

The band gap energy was determined from absorption spectra using the Tauc plot. It assumes that the energy-dependent absorption coefficient α can be expressed by the following equation

$(\alpha \cdot h\nu)^{1/\gamma}=B(h\nu-E\mathrm{g})$, where h is the Planck constant, ν is the photon's frequency, Eg is the band gap energy, and B is a constant. The γ factor depends on the nature of the electron transition and is equal to 1/2 or 2 for the direct and indirect transition band gaps, respectively [S5]. Coefficient γ was determined according to the occurrence of a linear section on the Tauc plot (dependence of $(\alpha \cdot h\nu)^{1/\gamma}$ on photon energy).

Obtained results were used for creation of Fig. 6 (positions of VB and CB).

1.3.7. Fermi level position determination and VB, CB bands alignment.

The positions of the Fermi level (E_F) of the semiconductors were determined from the positions of the corresponding valence bands, using the standard procedure of extrapolating the edge(s) of the XPS spectra to its intersection with the background, which corresponds to the difference between the valence level (E_V) and the Fermi level (E_F).

In order to elucidate the alignment of the valence and conduction bands and in turn to confirm the mechanism of the overall water splitting, additional XPS measurements and subsequent calculations were accomplished according to previously published works [S8-S10].

The valence band offset ΔE_V was calculated according to equation:

$$\Delta E_V = (E^{WO_3}_{W\,4f_{\frac{7}{2}}} - E^{WO_3}_{VBM}) - (E^{CdS}_{Cd\,3d_{\frac{5}{2}}} - E^{CdS}_{VBM}) - (E^{*\,WO_3}_{W\,4f_{\frac{7}{2}}} - E^{*\,CdS}_{Cd\,3d_{\frac{5}{2}}}),$$

where $E^{WO_3}_{W\,4f_{\frac{7}{2}}}$ and $E^{CdS}_{Cd\,3d_{\frac{5}{2}}}$ are core-level energies for W $4f_{7/2}$ of WO_3 and Cd $3d_{5/2}$ of CdS respectively; $E^{WO_3}_{VBM}$ and E^{CdS}_{VBM} are the values of the valence band maximum (VBM) measured for WO_3 and CdS respectively; $E^{*\,WO_3}_{W\,4f_{\frac{7}{2}}}$ and $E^{*\,CdS}_{Cd\,3d_{\frac{5}{2}}}$ are the values of core-level energies for W $4f_{7/2}$ and Cd $3d_{5/2}$ of WC-700 sample.

Then the conductive band offset ΔE_C was calculated as follows:

$$\Delta E_C = \Delta E_V - E^{WO_3}_{gap} + E^{CdS}_{gap},$$

where $E^{WO_3}_{gap}$ and E^{CdS}_{gap} are bandgaps for WO_3 and CdS respectively.

Obtained results were used for creation of Fig. 6 (position of Fermi level and bands alignment).

Table S1 XPS measured elements concentration (in at. %) on the surface of WO_3/CdS flakes annealed at different temperature.

	**Surface elements concentration** (at. %)			
	**W**	**O**	**Cd**	**S**
**W-0**	**30.1**	**69.9**	**-**	**-**
**WC-0**	**25.4**	**68.7**	**3.0**	**2.9**
**WC-300**	**31.0**	**64.5**	**2.2**	**2.3**
**WC-500**	**24.8**	**69.8**	**2.9**	**2.5**
**WC-600**	**21.3**	**73.5**	**3.3**	**1.9**
**WC-700**	**20.5**	**74.6**	**3.6**	**1.3**
**WC-800**	**28.3**	**68.6**	**2.6**	**0.5**
**WC-900**	**64.7**	**35.2**	**< 0.1**	**< 0.1**

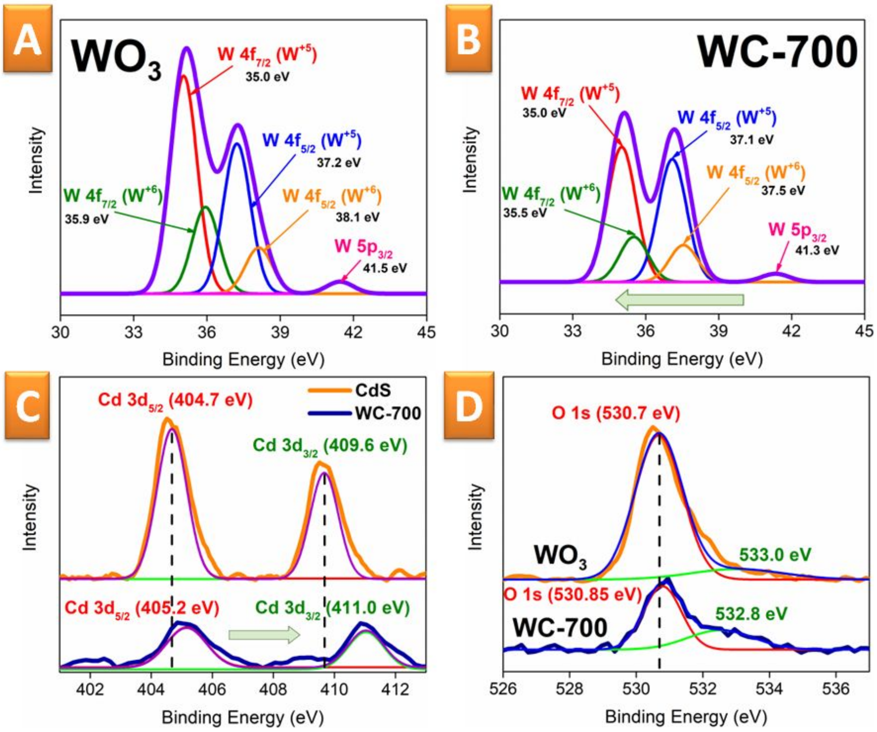

Fig. S1 Fittings of high-resolution XPS spectra - regions corresponding to W (in the structure of WO_3 (A) and WC-700 (B) samples); (C) - regions corresponding to Cd (in the structure of separately

prepared CdS and WC-700 samples) and (D) - regions corresponding to O (in the structure of WO_3 and WC-700 samples).

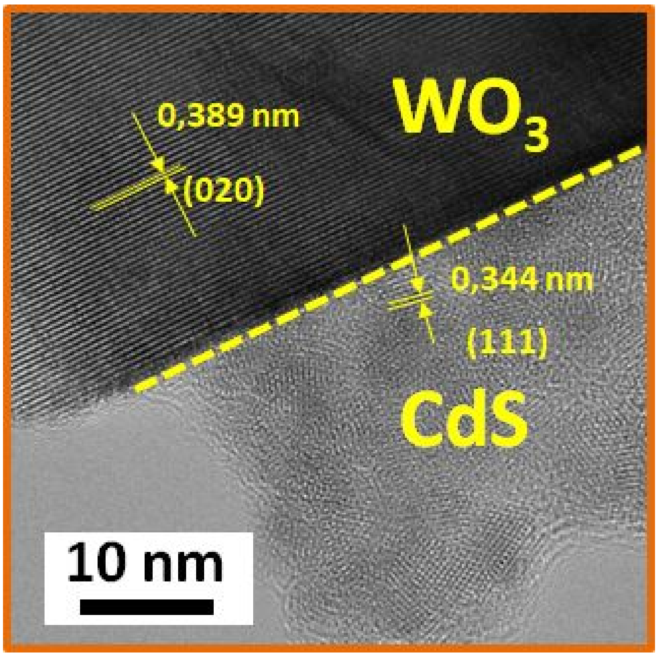

Fig. S2 HRTEM image of pristine WO_3/CdS flake.

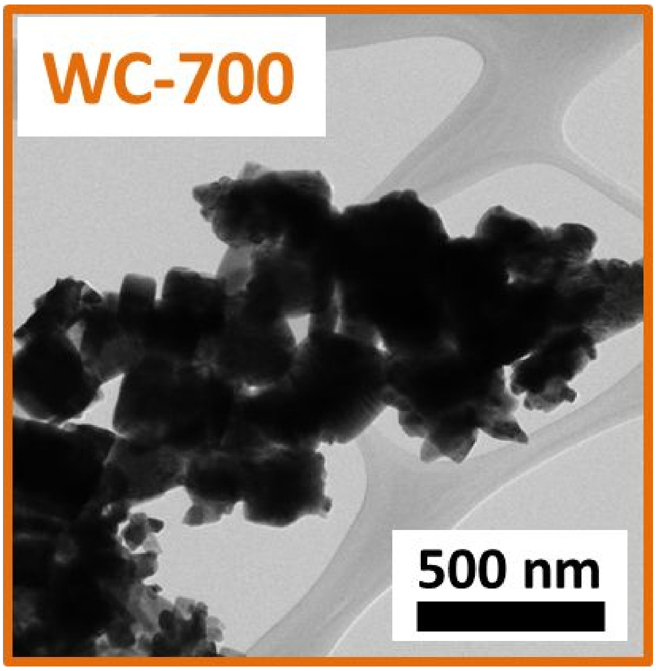

Fig. S3 TEM image of WC-700 flakes

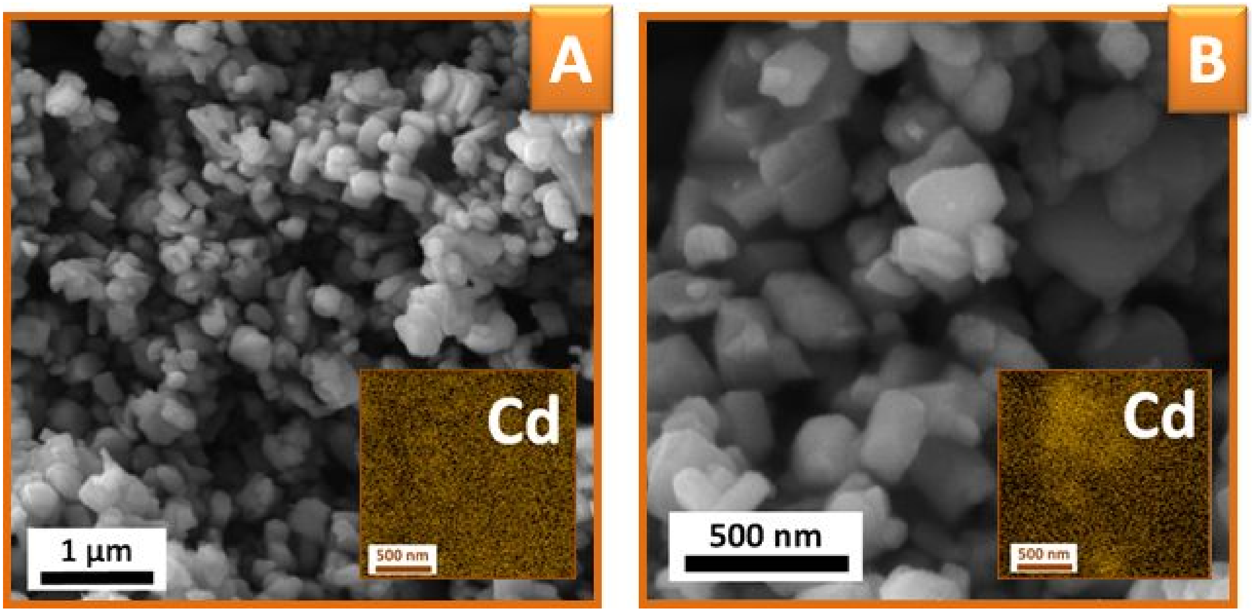

Fig. S4 SEM images together with Cd EDX mappings of WC-0 (A) and WC-700 (B) nanostructures.

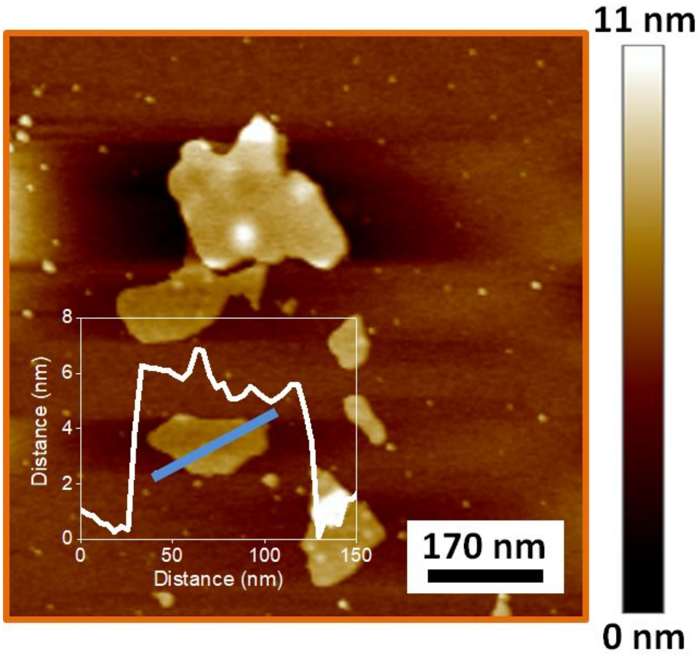

Fig. S5 AFM image of pristine WC-0 flakes and corresponding thickness profile of typical flake.

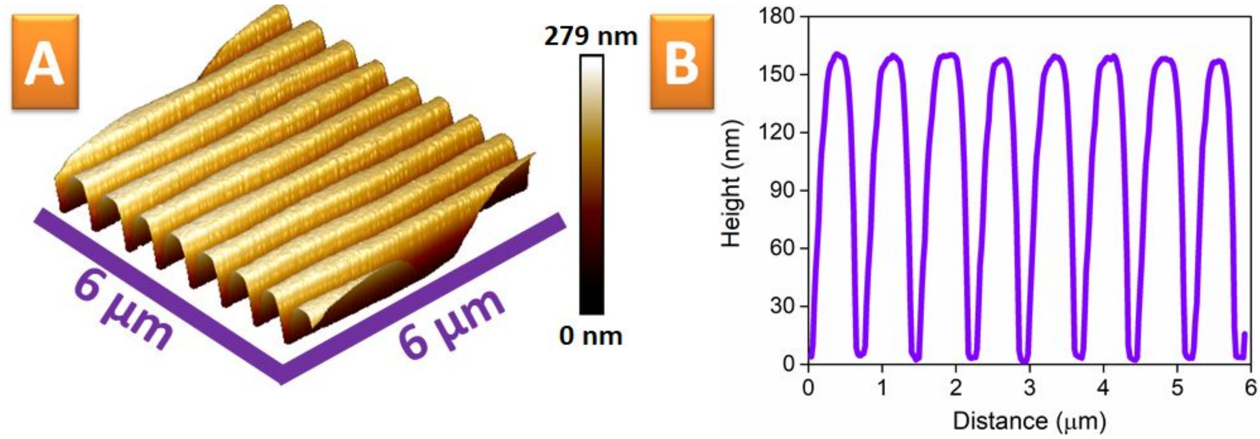

Fig. S6 AFM image of pristine Au grating (A) and its surface profile (B).

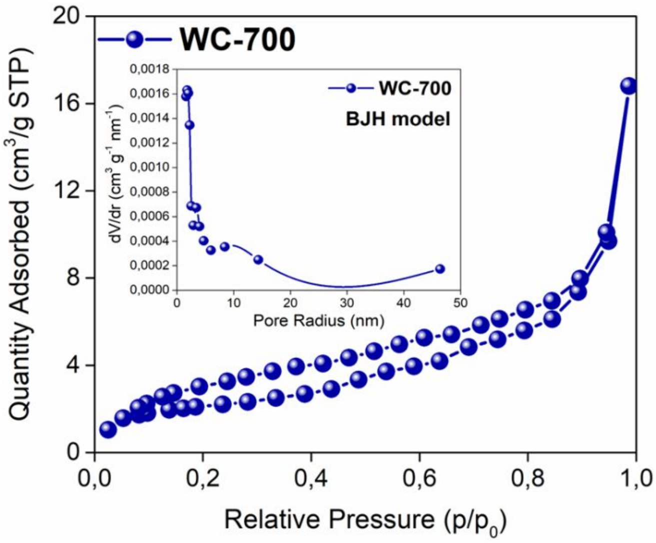

Fig. S7 N_2 adsorption/desorption isotherms, measured on WC-700 powder, and evaluated in BJP model pore size distribution.

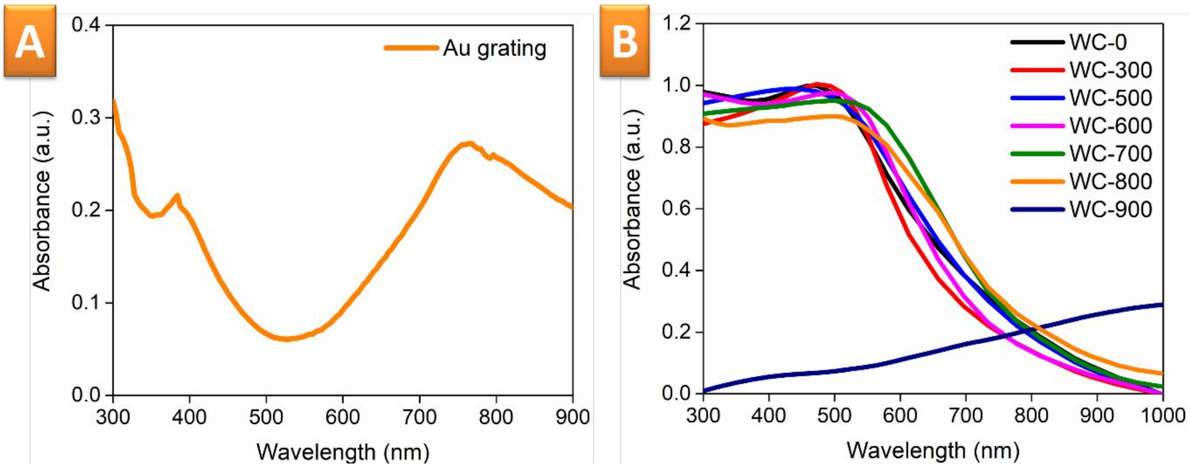

Fig. S8 (A) - UV-Vis absorption spectrum of Au grating; (B) - UV-Vis absorption spectra of WC-X flakes as a function of annealing temperature.

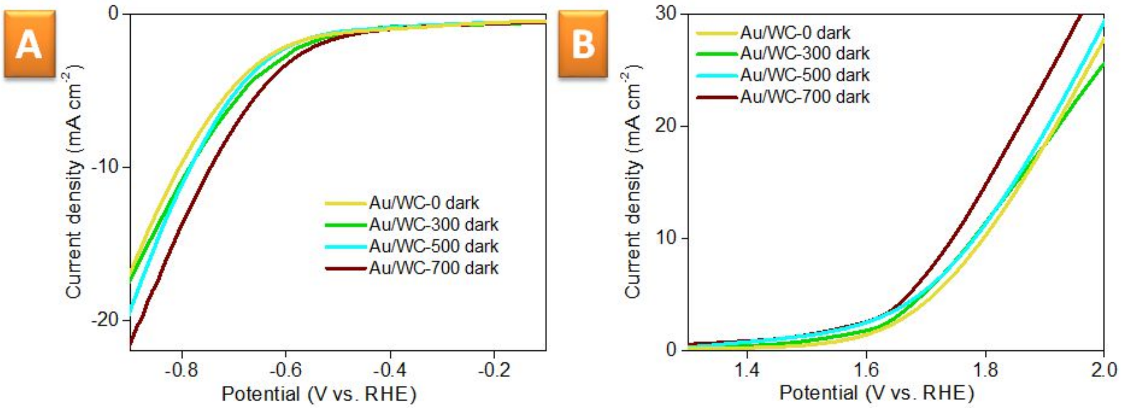

Fig. S9 LSV curves measured in dark in HER (A) or OER (B) potentials on Au/WC-X electrode surface as a function of annealing temperature.

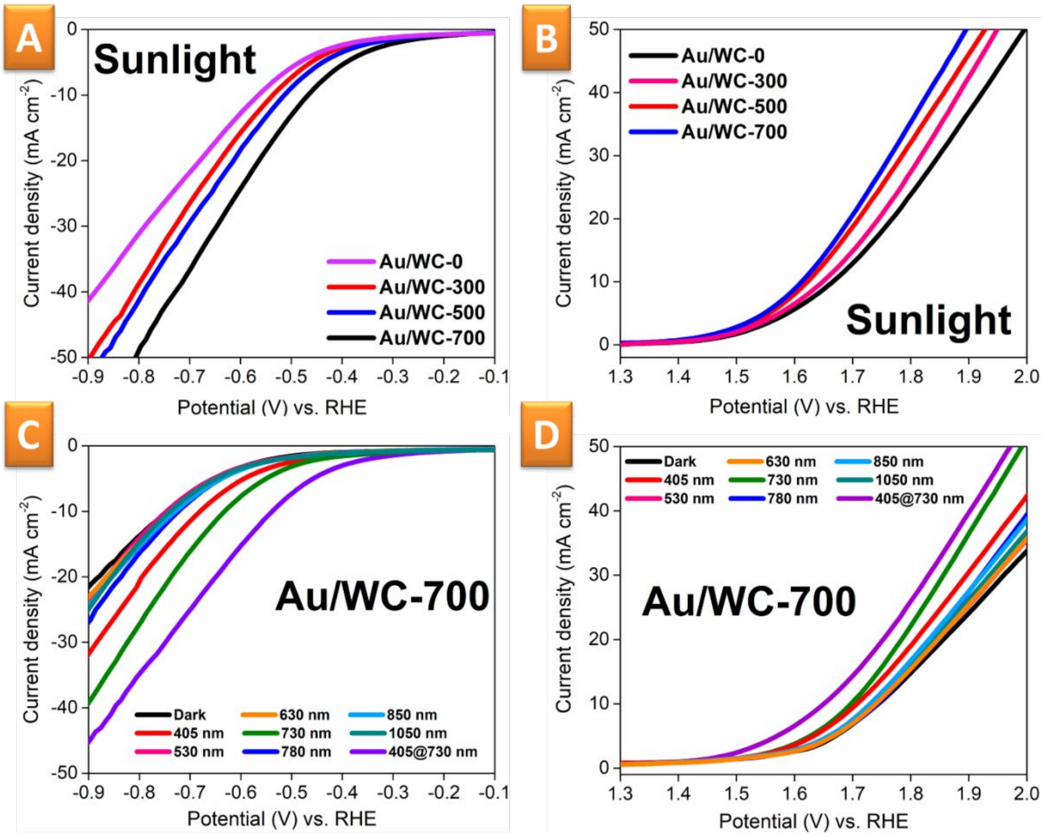

Fig. S10 LSV curves measured in dark or under illumination in HER or OER potentials on Au/WC-X photoelectrode surface: (A), (B) – impact of WO_3/CdS annealing temperature on the shift of LSV curve (photoelectrochemical regime); (C), (D) – impact of illumination wavelengths on HER or OER photoelectrochemical efficiency on Au/WC-700 surface.

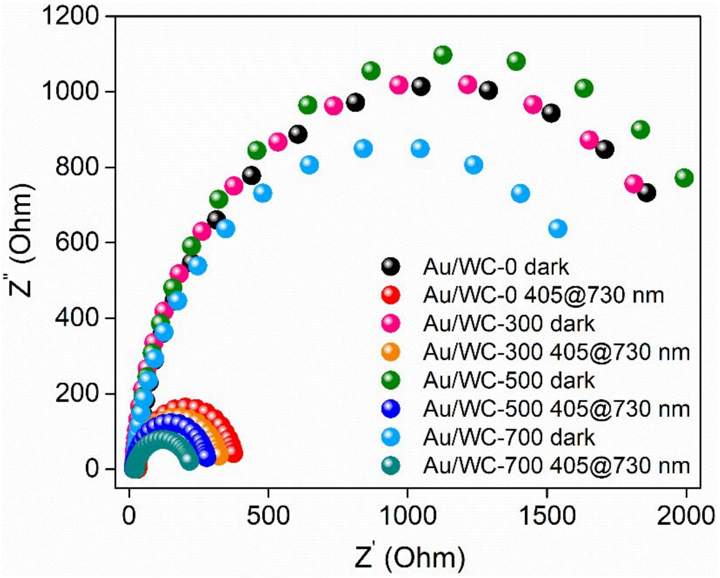

Fig. S11 EIS spectra, measured as a function of samples previous annealing in dark or under light illumination.

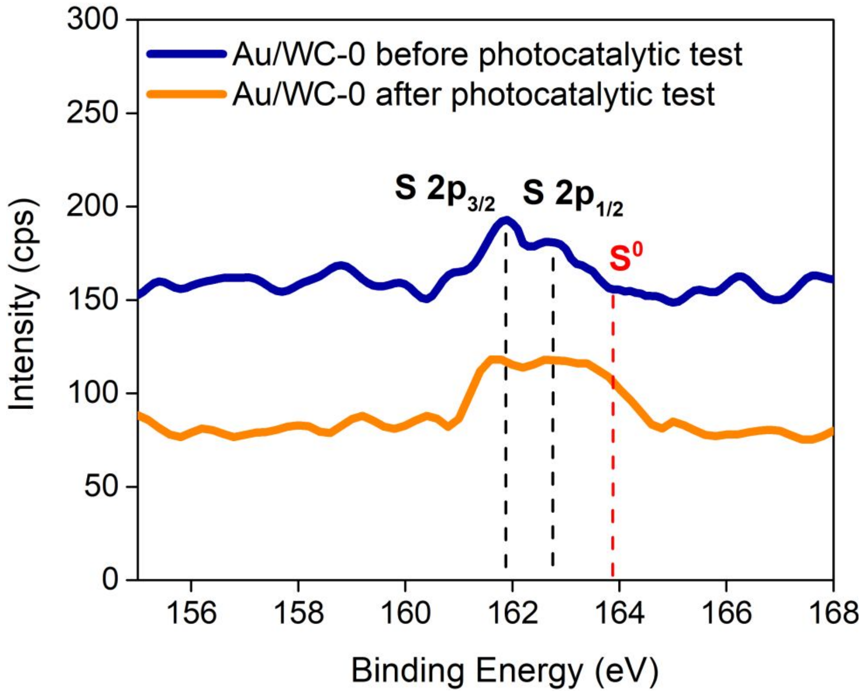

Fig. S12 Details of XPS S 2p peak, measured on WC-0 before (blue) and after (red) illumination in water for 10 hours). The peak change demonstrates illumination-induced sulphur oxidation in the case of WC-0 sample used in water splitting test

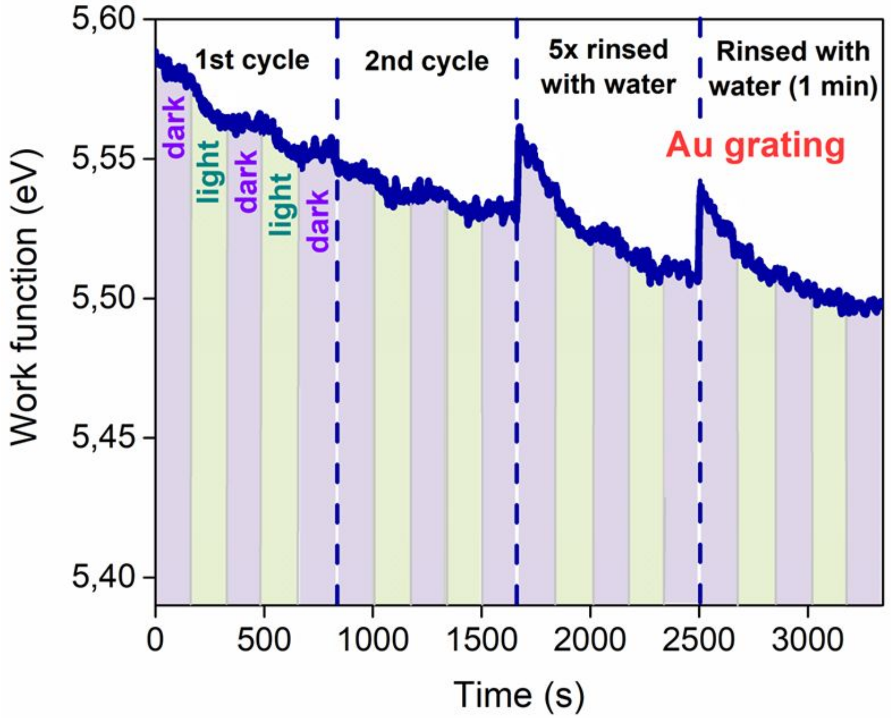

Fig. S13 Work function, measured on Au grating surface as a function of sample illumination switching ON/OFF or rinsing with water.

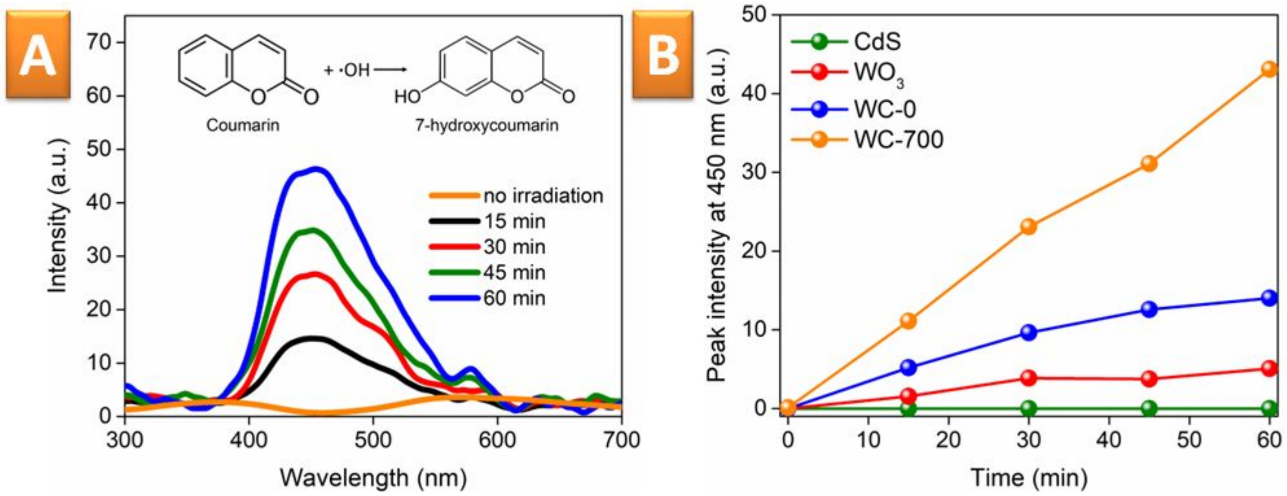

Fig. S14 (A) – PL spectra of 7-hydroxycoumarin, created after irradiation of WC-700 suspension in the presence of coumarin; (B) - PL peak intensity as a function of the illumination time and semiconductor(s) used for radicals generation and 7-hydroxycoumarin production.

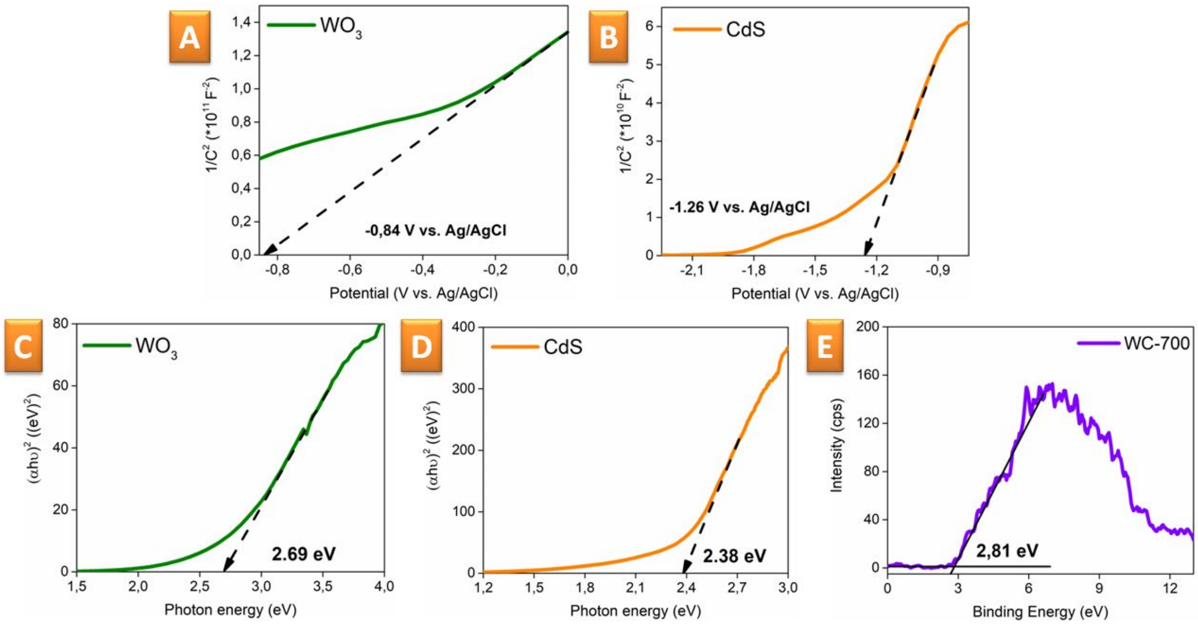

Fig. S15 (A), (B) - Mott–Schottky plots of WO_3 and CdS; (C), (D) – Tauc plots of WO_3 and CdS; (E) – XPS valence band spectrum of WC-700 sample.

References

(S1) Zabelin, D.; Zabelina, A.; Tulupova, A.; Elashnikov, R.; Kolska, Z.; Svorcik, V.; Lyutakov, O. A Surface Plasmon Polariton-Triggered Z-Scheme for Overall Water Splitting and Solely Light-Induced Hydrogen Generation. *J. Mater. Chem. A* **2022**, *10* (26), 13829–13838. DOI: 10.1039/D2TA02365B.

(S2) Jin, J.; Yu, J.; Guo, D.; Cui, C.; Ho, W. A Hierarchical Z-Scheme CdS–WO_3 Photocatalyst with Enhanced CO_2 Reduction Activity. *Small* **2015**, *11* (39), 5262–5271. DOI: 10.1002/smll.201500926.

(S3) Verveniotis, E.; Kromka, A.; Rezek, B. Controlling Electrostatic Charging of Nanocrystalline Diamond at Nanoscale. *Langmuir* **2013**, *29* (23), 7111–7117. https://doi.org/10.1021/la4008312.

(S4) Čermák, J.; Koide, Y.; Takeuchi, D.; Rezek, B. Spectrally Dependent Photovoltages in Schottky Photodiode Based on (100) B-Doped Diamond. *J. Appl. Phys.* **2014**, *115* (5), 053105. https://doi.org/10.1063/1.4864420.

(S5) Pankove, J. I. *Optical Processes in Semiconductors*; Courier Corporation, 1975.

(S6) Bai, S.; Jiang, J.; Zhang, Q.; Xiong, Y. Steering Charge Kinetics in Photocatalysis: Intersection of Materials Syntheses, Characterization Techniques and Theoretical Simulations. *Chem. Soc. Rev.* **2015**, *44* (10), 2893–2939. https://doi.org/10.1039/C5CS00064E.

(S7) Yin, W.; Bai, L.; Zhu, Y.; Zhong, S.; Zhao, L.; Li, Z.; Bai, S. Embedding Metal in the Interface of a P-n Heterojunction with a Stack Design for Superior Z-Scheme Photocatalytic Hydrogen Evolution. *ACS Appl. Mater. Interfaces* **2016**, *8* (35), 23133–23142. https://doi.org/10.1021/acsami.6b07754.

(S8) Iqbal, A.; Kafizas, A.; Sotelo-Vazquez, C.; Wilson, R.; Ling, M.; Taylor, A.; Blackman, C.; Bevan, K.; Parkin, I.; Quesada-Cabrera, R. Charge Transport Phenomena in Heterojunction Photocatalysts: The WO_3/TiO_2 System as an Archetypical Model. *ACS Appl. Mater. Interfaces* **2021**, *13* (8), 9781–9793. https://doi.org/10.1021/acsami.0c19692.

(S9) Rosa, W. S.; Rabelo, L. G.; Tiveron Zampaulo, L. G.; Gonçalves, R. V. Ternary Oxide $CuWO_4/BiVO_4/FeCoO_x$ Films for Photoelectrochemical Water Oxidation: Insights into the Electronic Structure and Interfacial Band Alignment. *ACS Appl. Mater. Interfaces* **2022**, *14* (20), 22858–22869. https://doi.org/10.1021/acsami.1c21001.

(S10) Nogueira, A. C.; Gomes, L. E.; Ferencz, J. A. P.; Rodrigues, J. E. F. S.; Gonçalves, R. V.; Wender, H. Improved Visible Light Photoactivity of $CuBi_2O_4/CuO$ Heterojunctions for Photodegradation of Methylene Blue and Metronidazole. *J. Phys. Chem. C* **2019**, *123* (42), 25680–25690. https://doi.org/10.1021/acs.jpcc.9b06907.
